# Supplementary material for: Gene flow improves fitness at a range edge under climate change
Source: Evol Lett. 2018 Nov 30;3(1):55–68. doi: 10.1002/evl3.91 (PMC6369935; doi:10.1002/evl3.91)
Supplement: Supplementary file 1 — Table S1. Geographic information for the populations of Clarkia pulchella used in this experiment. Table S2. Results of generalized linear mixed effects models for the effect of local vs. foreign origin on performance of Clarkia pulchella in common gardens. Table S3. Results of generalized linear mixed effects models of the effects of absolute precipitation and temperature differences on component lifestages of Clarkia pulchella. Table S4. Results of generalized linear mixed effects models of the effects of being a within‐population cross vs. a between‐population cross, while accounting for effects of absolute precipitation and temperature differences. Table S5. Results of generalized linear mixed effects models separately testing the effects of (A) genetic differentiation, (B) absolute midparent temperature differences, and (C) absolute midparent precipitation differences on performance of Clarkia pulchella in common gardens. Table S6. Results of generalized linear mixed effects models of the effects of genetic differentiation between parental populations on performance of Clarkia pulchella in common gardens. Table S7. Results of generalized linear mixed effects models for the effect of local vs. foreign origin on performance of Clarkia pulchella including covariates of absolute precipitation and temperature differences (Supporting information Analysis 2). Figure S1. Correlation of temperature and precipitation normals calculated over the years 1951‐1980 and 1984‐2013. Figure S2. Temperature and precipitation normals calculated over the years 1951‐1980 and 1984‐2013, shown paired by site. Figure S3. Distribution of climate differences of within‐ vs. between‐population crosses of Clarkia pulchella relative to conditions during the experiment. Figure S4. Lifetime fitness (seeds produced per seed planted) from populations of Clarkia pulchella with foreign vs. local parents. Figure S5. Regression estimates and predicted lifetime fitness of local populations (black) com [file EVL3-3-55-s001.pdf]

Supplementary material for "Gene flow improves fitness at a range edge under climate change"

Megan Bontrager[1,3] and Amy L. Angert[2]

[1]Department of Botany, University of British Columbia, Vancouver, British Columbia V6T 1Z4, Canada

[2]Departments of Botany and Zoology, University of British Columbia, Vancouver, British Columbia V6T 1Z4, Canada

[3]Author for correspondence (mgbontrager@gmail.com)

Supplementary text

Supplementary methods **1**

1. Greenhouse conditions and crossing design 2
2. Transplant installation 2
3. Details of monitoring and measuring 3
4. Ovary length as a fitness proxy 4

Supplementary analyses and results **6**

1. Do warmer foreign populations outperform local populations? 6
2. Are there benefits of being local once climate of origin is controlled for? 6

Supplementary tables

S1 Geographic information for populations used in the transplant experiment 8
S2 Effects of local vs. foreign origin 9
S3 Effects of temperature and precipitation differences 10
S4 Effects of being a within-population cross vs. a between-population cross 11
S5 Separate tests of effects of genetic differentiation, temperature differences, and precipitation differences 12
S6 Effects of genetic differentiation between parental populations 13
S7 Effects of local vs. foreign origin with climate covariates included 14

Supplementary figures

S1 Correlation of historic and recent climate normals 15
S2 Historic and recent climate normals paired by site 16
S3 Climate differences of within vs. between population crosses 17
S4 Local populations vs. foreign populations 18
S5 Local populations compared to warmer and cooler foreign populations 19
S6 Ovary length correlates with seed set 20

Supplementary methods

1. Greenhouse conditions and crossing design

Seeds were planted in the greenhouse 9-11 December, 2014 in conetainers (Stuewe and Sons, Tangent, Oregon, USA) filled with Sunshine Mix No. 4 (Sun Gro Horticulture, Agawam, MA, USA). For each of 22 maternal families per population, 3-5 seeds were planted on the soil surface in each pot. For families from each of the two focal populations, three replicate pots were planted per family because larger quantities of flowers would be needed from these families; other populations were represented by one replicate per family. Pots were arranged into randomized blocks, with each block containing one family from each population (one pot from each donor population and three replicate pots from each of the two focal populations). The soil was kept moist until germination, then plants were hand watered every 1-3 days as needed to prevent wilting. After germination, plants were thinned randomly to one per cone and pumice was added to the soil surface to prevent fungal growth. Plants began to flower in March 2015. Plants were bagged to prevent unintentional pollination, and flowers were emasculated upon opening to prevent self-pollination. For the crosses, 20 of the 22 blocks were used, the other two were maintained in the same growing conditions to provide alternate plants in case of mortality or sterility. We performed as many crosses as possible using a single focal plant, but if flower production was too low on that plant we also used one of the replicate focal plants from the same family. Most crosses had to be performed 2-3 times to obtain adequate numbers of seeds for the experiment. Some crosses could not be performed due to mortality, sterility, or limited flower production. As ripening progressed, the ends of fruits were taped shut to prevent seed loss. Upon ripening, fruits were collected and stored in coin envelopes in the lab. Crosses were performed March-May 2015 and and we collected fruits March-June 2015.

2. Transplant installation

Seeds attached to toothpicks were planted in the ground 18-21 September 2015. Plots were prepared by removing litter, large rocks, and dried remains of herbaceous perennial plants. The ground surface was minimally leveled to allow for placement of planting grids that aided in consistently spacing the plants. Each toothpick was inserted into the ground gently so that seeds were not dislodged or

damaged until seeds were ~3 mm below the soil surface. Toothpicks were inserted at 5 cm spacing into ~1 m by 2 m blocks. Block shape was varied to accommodate rocks and shrubs surrounding the planting area. After planting, each block was protected with 20 cm high hardware cloth cages supported by rebar. These cages were intended to prevent trampling by larger animals but did not prevent entry of rodents and other small animals. The area surrounding the plots at each site was sprayed with deer repellent several times during the course of the experiment. To ensure germination, plots were watered at a rate of ~10 L per plot 27-29 October 2015, though at that time most seeds that were checked already had radicles emerging. In May 2016 cattle fencing was put around the plots at the Blue Lake site before cattle were released into the area for grazing; this fencing succeeded in keeping the cows off the plots. No cattle were present at the Rock Creek site.

3. Details of monitoring and measuring

Our transplant gardens were installed in areas where *C. pulchella* occurs naturally, and because of this we wanted to evaluate how frequently we might have mistaken naturally occurring plants for experimental plants. During germination surveys we censused one more germinant than the number of seeds that we planted at 23 out of 16,680 grid points (0.14%). This gives an estimate of the minimum rate at which naturally occurring seeds were indistinguishable from our planted seedlings. So, while it is probable that some naturally occurring plants were mistaken for experimental plants, we consider the frequency of possible misidentification to be acceptably low.

During winter, some plots were affected by frost-heave and seedlings were uprooted from their planting locations when their toothpick was forced out of the ground (1901/16680 grid points, 11.4%). In lightly affected areas, toothpicks and seedlings were gently settled back into the soil. In more heavily affected areas, individual identity could no longer be determined confidently and individuals were excluded from further measurements and analyses (95/16680 grid points, 0.57%).

Censuses of reproduction began on 2 June 2016. On 12-13 June 2016 we censused spring survival of all plants. Once flowering began, we placed bridal-veil nets over the hardware cloth on each plot to prevent pollen escape into local populations. In June we censused each plot every 2-3 days. We recorded the date of first flowering of each plant at this temporal resolution. During each census,

the immature ovary length of each new flower was recorded to be used as a proxy for maximum seed set. Flowers were marked as they were measured with a permanent marker and a running flower count was kept for each plant to avoid double-counting as flowers senesced. We continued these assessments as flowering slowed in July, but reduced the census interval to once a week. Damage to plants, such as rodent activity or herbivory, was noted during monitoring. Any plants with uncertain identities (due to frost damage as mentioned above, being far from their toothpick, or the toothpick disappearing; n = 201/16680 toothpicks, 1.2%) were excluded from all analyses. Plants that were killed by gophers, browsers, or galling insects were excluded from analyses that involved lifestages downstream from these events (n = 525/16479 plants, 3.2%) because we do not think that this mortality is related to population origin but rather to block-specific factors.

4. Ovary length as a fitness proxy

Pollinations were performed on a subset of plants to calibrate a conversion from immature ovary lengths to seed production. On 596 flowers (mean = 29.8 per plot, range = 0–126) stigmas were dusted with an ample pollen load using all four anthers from another plant in the plot. These flowers were marked with strings around the pedicles and fruits were collected when ripe. Seeds in each of these pollinated fruits were later counted in the lab. Total seed production per individual was estimated by multiplying the total ovary length of each plant by the average number of seeds per mm of immature ovary, as determined from the pollinated fruits. This resulted in an estimate of 4.75 seeds per mm based on a linear regression of number of seeds predicted by ovary length with the intercept set to 0 (Figure S6, $R_0^2 = 0.87$, $P < 0.0001$, n = 596). This may be an over- or underestimate if our hand pollinations are more effective or less effective than natural pollinator service. For comparison, we measured ovary length and later seed set on a small number of naturally occurring *C. pulchella* plants near our plots (n = 73); the relationship between ovary length and number of seeds is similar in hand and natural pollinations (Figure S6).

We pollinated only a maximum of one flower per plant, so these may be overestimates because they do not account for potential resource limitation of seed set. If plants are prevented from setting seed due to pollen limitation, they may produce more flowers as a response. We found this to be

the case to a small extent in natural populations of *Clarkia pulchella*, where pollinator exclusion led to plants producing an average of 0.6 more fruits compared to controls (Bontrager et al., 2018). However, we checked whether variation in seeds per mm of fruit in our transplanted individuals was associated with individual fitness (the overall fruit production per individual) or block quality (which we estimated based on the average fruit production of a block), and we could not attribute variation in seeds per mm of fruit to either of these factors. Therefore, while our conversion from fruit length to seeds may not be exact, we do not expect it to be systematically biased by plant size or resource availability.

An ANOVA indicated a significant effect of source population on seeds per mm of fruit for within-population plants ($P = 0.040$, $F_{14,298} = 1.79$) and a nearly significant effect for between-population plants ($P = 0.060$, $F_{14,298} = 1.68$). However, posthoc comparisons (Tukey HSD) did not reveal any significant pairwise differences. So while it is possible that there is interesting variation among populations in seed size or ovary morphology, we were not confident that we would be able to characterize this variation in sources where our hand pollination sample size is low, so we elected to use the same conversion across all populations.

We also tested whether seeds per mm of fruit covaried with any of the predictor variables that we used in our analyses, such that our conversion might amplify or dampen climate-fitness or climate-gene flow relationships. We found a small but significant difference between within-population and between-population plants in seeds per mm of fruit (within-population plants produced 0.8 fewer seeds per mm, on average; $P < 0.0001$). As a result, we may have slightly underestimated the positive effects of gene flow on seed production. We also found a small but significant effect of historic precipitation of each source population on seeds per mm of fruit, with populations from the driest site in the experiment producing on average 2.14 more seeds per mm of fruit than those from the wettest site ($P < 0.0001$). This could reflect either adaptive differentiation in seed size in response to precipitation, or that populations that were best matched to the experiment conditions (those from dry sources) not only made larger fruits but also produced more seeds per mm of fruit. In this case, our use of ovary length as a proxy for seed set is conservative, as it might have dampened the fitness benefits of being well matched to precipitation, rather than inflating them.

We found no effects of temperature of origin or F_{ST} on seeds produced per mm of fruit.

Supplementary analyses and results

1. Do warmer foreign populations outperform local populations?

Our main analyses indicate that populations from historic climates better matched to the experimental conditions performed best. This raises the question of whether foreign populations from warmer sites outperform local populations when they are compared directly (as well as whether cooler foreign populations perform worse than local populations). To test this, we designated foreign populations as either warmer or cooler than local populations by comparing their historic temperature normals to the historic temperature normals of our focal populations. We then ran a zero-inflated negative binomial GLMM comparing the lifetime fitness of local populations, warmer foreign populations, and cooler foreign populations. We included random effects of site within block and sire population (models with a more complex random effect structure would not converge).

We found that warmer foreign populations had a higher probability of producing seeds than the local populations (zero-inflation component: $\beta = 0.344$, SE = 0.126, $P = 0.006$, Figure S5A; conditional component: $\beta = 0.153$, SE = 0.136, $P = 0.260$, Figure S5B). Similarly, cooler foreign populations had a lower probability of producing seeds than the local populations (zero-inflation component: $\beta = -0.316$, SE = 0.129, $P = 0.015$, Figure S5A; conditional component: $\beta = 0.000$, SE = 0.136, $P = 0.999$, Figure S5B). These results support the inference that temperature of origin is an important driver of performance, and show that this leads to categorical differences between local, warmer foreign, and cooler foreign populations, (Figure S5C).

2. Are there benefits of being local once climate of origin is controlled for?

If the focal populations in this experiment are locally adapted to conditions other than the climate variables that we have considered here, they may have fitness advantages over foreign populations once climate of origin has been accounted for. To test this, we re-ran our analyses of local vs. foreign performance and included absolute temperature and precipitation differences as covariates

(essentially combining the first two analyses described in the main text methods). Our statistical methods were the same as in our main analyses: for lifetime fitness responses we used a GLMM with a zero-inflated negative binomial distribution. We also built individual models of component lifestages in which we used climate data from only the months in each census window. We did not find any significant differences between local and foreign fitness, even once the effects of climate were accounted for (Table S7).

References

Bontrager, M., C. D. Muir, and A. Angert (2018). Geographic and climatic drivers of reproductive assurance in *Clarkia pulchella*. *bioRxiv*, 372375.

PRISM Climate Group (2017). Oregon State University. Accessed: 2017-09-30.

Supplementary tables and figures

Table S1 Geographic information for the populations of *Clarkia pulchella* used in this experiment.

Population name	Map ID	Type	Latitude	Longitude	Elevation (m)
Blue Lake	F1	Focal	49.05	-119.56	842
Johnstone Creek	F2	Focal	49.04	-119.05	866
Border	D1	Donor	48.98	-118.99	1211
Day Creek Road	D2	Donor	48.94	-118.51	911
Bodie Mountain	D3	Donor	48.83	-118.83	1603
Boulder Creek Road	D4	Donor	48.76	-118.33	1115
Aeneas Valley	D5	Donor	48.54	-118.91	1126
Henry Creek Road	D6	Donor	47.45	-114.77	1103
Heyburn State Park	D7	Donor	47.34	-116.79	801
McCrosky State Park	D8	Donor	47.09	-116.98	1186
Graves Creek Road	D9	Donor	46.80	-114.41	1201
Bitterroot	D10	Donor	46.54	-113.89	1424
Abel's Ridge	D11	Donor	46.28	-117.60	1457
Tucannon	D12	Donor	46.24	-117.74	1022
Pendleton	D13	Donor	45.74	-118.25	649

Table S2 Results of generalized linear mixed effects models for the effect of local vs. foreign origin on performance of *Clarkia pulchella* in common gardens. There are no significant differences between populations of local vs. foreign origin in fitness components or lifetime fitness. Size during the previous census (November for overwinter survival and size, March for fruit counts and estimated seed production) is always a significant predictor of performance in subsequent lifestages.

Response	Foreign vs. local origin			Size during previous census		
	Estimate	SE	P-value	Estimate	SE	P-value
Germination	-0.034	0.088	0.702	-	-	-
Size after germination	0.027	0.073	0.708	-	-	-
Overwinter survival	0.176	0.126	0.161	**0.377**	**0.033**	$<$ **0.001**
Size after winter	-0.068	0.182	0.708	**0.750**	**0.042**	$<$ **0.001**
Fruit count	0.089	0.146	0.544	**0.544**	**0.041**	$<$ **0.001**
Seed production	-0.005	0.128	0.966	**0.388**	**0.032**	$<$ **0.001**
Lifetime fitness - zero inflation	-0.032	0.123	0.798	-	-	-
Lifetime fitness - conditional	-0.093	0.128	0.469	-	-	-

Table S3 Results of generalized linear mixed effects models of the effects of absolute precipitation and temperature differences on component lifestages of *Clarkia pulchella*. Temperature and precipitation differences refer to absolute differences between the historic conditions that a population experienced and the conditions in the common gardens during the experiment. These differences were calculated using climate data from only the months of that census period (i.e., September-November for germination and size after germination, December-March for overwinter survival and size after winter, and April-July for fruit counts and seed production). Analyses were conducted using only plants surviving the previous census window. Whenever applicable, size in the previous census was included as a covariate to account for differences accumulated during earlier lifestages. Significant parameters are indicated with bold text. These results are visually summarized in Figure 3.

	Absolute temperature difference			Absolute precipitation difference			Size in previous census		
Response	Estimate	SE	P-value	Estimate	SE	P-value	Estimate	SE	P-value
Germination	**-0.094**	**0.035**	**0.007**	-	-	-	-	-	-
Size after germination	-0.027	0.026	0.293	-	-	-	-	-	-
Overwinter survival	**-0.078**	**0.032**	**0.015**	-	-	-	**0.366**	**0.033**	$<$ **0.001**
Size after winter	**-0.518**	**0.095**	$<$ **0.001**	-	-	-	**0.748**	**0.042**	$<$ **0.001**
Fruit count	-0.114	0.071	0.108	-0.091	0.058	0.119	**0.543**	**0.041**	$<$ **0.001**
Seed production	-0.055	0.047	0.249	**-0.110**	**0.044**	**0.011**	**0.389**	**0.033**	$<$ **0.001**

Table S4 Results of generalized linear mixed effects models of the effects of being a within-population cross vs. a between-population cross, while accounting for effects of absolute precipitation and temperature differences. Temperature and precipitation differences refer to absolute differences between the average historic conditions of an individual's parental populations and the conditions in the common gardens during the experiment. Positive estimates of the effects of between-population vs. within-populations indicate that having parents from two different populations ("gene flow") is beneficial. **(A)** Effects on lifetime fitness of *Clarkia pulchella*. **(B)** Effects on lifetime fitness when midparent precipitation differences are not included in the model. **(C)** Effects on component lifestages. Climate differences were calculated using climate data from only the months of that census period (i.e., September-November for germination and size after germination, December-March for overwinter survival and size after winter, and April-July for fruit counts and seed production). Analyses were conducted using only plants surviving the previous census window. Whenever applicable, size in the previous census was included as a covariate to account for differences accumulated during earlier lifestages. Significant parameters are indicated with bold text. These results are visually summarized in Figure 4.

	Between-populations vs. within-populations			Absolute midparent temperature difference			Absolute midparent precipitation difference			Size during previous census		
Response	Estimate	SE	*P*-value	Estimate	SE	*P*-value	Estimate	SE	*P*-value	Estimate	SE	*P*-value
**A. Lifetime fitness**												
Lifetime fitness - zero inflation	0.008	0.047	0.860	**-0.240**	**0.023**	$<$ **0.001**	-	-	-	-	-	-
Lifetime fitness - conditional	0.047	0.049	0.345	**-0.085**	**0.032**	**0.007**	**-0.072**	**0.036**	**0.045**	-	-	-
**B. Lifetime fitness without precipitation in model**												
Lifetime fitness - zero inflation	0.008	0.047	0.860	**-0.240**	**0.023**	$<$ **0.001**	-	-	-	-	-	-
Lifetime fitness - conditional	**0.114**	**0.036**	**0.002**	**-0.059**	**0.029**	**0.041**	-	-	-	-	-	-
**C. Component lifestages**												
Germination	-0.035	0.078	0.653	**-0.088**	**0.029**	**0.003**	-	-	-	-	-	-
Size after germination	-0.033	0.041	0.421	-0.028	0.015	0.058	-	-	-	-	-	-
Overwinter survival	0.012	0.047	0.794	**-0.058**	**0.022**	**0.009**	-	-	-	**0.336**	**0.023**	$<$ **0.001**
Size after winter	-0.052	0.127	0.678	**-0.274**	**0.068**	$<$ **0.001**	-	-	-	**0.721**	**0.031**	$<$ **0.001**
Fruit count	**0.125**	**0.055**	**0.021**	**-0.081**	**0.032**	**0.011**	**-0.092**	**0.034**	**0.007**	**0.493**	**0.030**	$<$ **0.001**
Seed production	0.092	0.047	0.052	-0.030	0.028	0.285	**-0.092**	**0.030**	**0.002**	**0.357**	**0.024**	$<$ **0.001**

Table S5 Results of generalized linear mixed effects models separately testing the effects of **(A)** genetic differentiation, **(B)** absolute midparent temperature differences, and **(C)** absolute midparent precipitation differences on performance of *Clarkia pulchella* in common gardens. Absolute midparent temperature and precipitation differences refer to absolute differences between the conditions in the common gardens during the experiment and the average historic conditions of an individual's parental populations. These analyses were performed using between-population crosses only, that is, every plant has one parent from a focal population and one parent from a donor population. For analyses of lifetime fitness, temperature differences were calculated using the duration of the experiment and precipitation differences were calculated using summed April-July values. Precipitation differences are only included as an effect in the conditional part of the model of lifetime fitness because precipitation effects are expected to manifest at later lifestages. For analyses of component lifestages, climate differences were calculated using climate data from only the months of that census period (i.e., September-November for germination and size after germination, December-March for overwinter survival and size after winter, and April-July for fruit counts and seed production). Component lifestage analyses were conducted using only plants surviving the previous census window. Whenever applicable, size in the previous census was included as a covariate to account for differences accumulated during earlier lifestages. Significant parameters are indicated with bold text. These results are visually summarized in Figure 5.

A. F_{ST}

Response	F_{ST} Estimate	SE	P-value	Size during previous census Estimate	SE	P-value
Lifetime fitness - zero inflation	**0.086**	**0.028**	**0.002**	-	-	-
Lifetime fitness - conditional	**0.080**	**0.031**	**0.010**	-	-	-
Germination	**0.124**	**0.059**	**0.034**	-	-	-
Size after germination	0.014	0.014	0.306	-	-	-
Overwinter survival	0.054	0.035	0.124	**0.336**	**0.037**	**< 0.001**
Size after winter	**0.104**	**0.050**	**0.035**	**0.743**	**0.044**	**< 0.001**
Fruit count	**0.067**	**0.031**	**0.028**	**0.451**	**0.044**	**< 0.001**
Seed production	**0.081**	**0.029**	**0.005**	**0.334**	**0.035**	**< 0.001**

B. Absolute midparent temperature differences

Response	Temperature difference Estimate	SE	P-value	Size during previous census Estimate	SE	P-value
Lifetime fitness - zero inflation	**-0.148**	**0.046**	**0.001**	-	-	-
Lifetime fitness - conditional	-0.090	0.056	0.105	-	-	-
Germination	-0.088	0.070	0.212	-	-	-
Size after germination	-0.029	0.017	0.096	-	-	-
Overwinter survival	-0.054	0.049	0.275	**0.335**	**0.037**	**< 0.001**
Size after winter	-0.132	0.070	0.060	**0.743**	**0.044**	**< 0.001**
Fruit count	-0.121	0.063	0.057	**0.457**	**0.044**	**< 0.001**
Seed production	-0.071	0.066	0.285	**0.336**	**0.035**	**< 0.001**

C. Absolute midparent precipitation differences

Response	Precipitation difference Estimate	SE	P-value	Size during previous census Estimate	SE	P-value
Lifetime fitness - conditional	0.123	0.078	0.113	-	-	-
Fruit count	0.047	0.083	0.574	**0.452**	**0.044**	**< 0.001**
Seed production	0.099	0.076	0.192	**0.334**	**0.035**	**< 0.001**

Table S6 Results of generalized linear mixed effects models of the effects of genetic differentiation between parental populations on performance of *Clarkia pulchella* in common gardens. Effects of absolute precipitation and temperature differences are also included in these models. Temperature and precipitation differences refer to the absolute midparent differences, i.e., the absolute differences between the conditions in the common gardens during the experiment and the average historic conditions of an individual's parental populations. These analyses were performed using between-population crosses only, that is, every plant has one parent from a focal population and one parent from a donor population. **(A)** Effects on lifetime fitness. **(B)** Effects on component lifestages. Climate differences were calculated using climate data from only the months of that census period (i.e., September-November for germination and size after germination, December-March for overwinter survival and size after winter, and April-July for fruit counts and seed production). Analyses were conducted using only plants surviving the previous census window. Whenever applicable, size in the previous census was included as a covariate to account for differences accumulated during earlier lifestages. Significant parameters are indicated with bold text. These results are visually summarized in Figure 5.

	F_{ST}			Temperature difference			Precipitation difference			Size during previous census		
Response	Estimate	SE	*P*-value	Estimate	SE	*P*-value	Estimate	SE	*P*-value	Estimate	SE	*P*-value
**A. Lifetime fitness**												
Lifetime fitness - zero inflation	**0.064**	**0.030**	**0.030**	**-0.117**	**0.048**	**0.014**	-	-	-	-	-	-
Lifetime fitness - conditional	0.059	0.037	0.108	-0.031	0.055	0.574	0.053	0.082	0.520	-	-	-
**B. Component lifestages**												
Germination	0.111	0.062	0.073	-0.042	0.080	0.798	-	-	-	-	-	-
Size after germination	0.005	0.016	0.770	-0.026	0.020	0.179	-	-	-	-	-	-
Overwinter survival	0.047	0.044	0.286	-0.014	0.060	0.809	-	-	-	**0.335**	**0.037**	$<$ **0.001**
Size after winter	0.072	0.061	0.232	-0.073	0.082	0.376	-	-	-	**0.742**	**0.044**	$<$ **0.001**
Fruit count	0.058	0.034	0.089	-0.093	0.058	0.110	-0.010	0.084	0.905	**0.455**	**0.044**	$<$ **0.001**
Seed production	**0.071**	**0.033**	**0.031**	-0.035	0.055	0.522	0.028	0.077	0.719	**0.334**	**0.035**	$<$ **0.001**

Table S7 Results of generalized linear mixed effects models for the effect of local vs. foreign origin on performance of *Clarkia pulchella* including covariates of absolute precipitation and temperature differences (Supplementary Analysis 2). Temperature and precipitation differences refer to absolute differences between the historic conditions that a population experienced and the conditions in the common gardens during the experiment. Analyses of lifetime fitness **(A)** use temperature differences over the entire growing period (September-July) and precipitation differences during spring and summer (April-July). Analyses of component lifestages **(B)** use climate data from only the months of that census period (i.e., September-November for germination and size after germination, December-March for overwinter survival and size after winter, and April-July for fruit counts and seed production), and these analyses were conducted using only plants surviving the previous census window. Size during the previous census (November for overwinter survival and size, March for fruit counts and estimated seed production) was also included as a covariate to account for differences accumulated during earlier lifestages. Significant parameters are indicated with bold text.

	Foreign vs. local origin			Temperature difference			Precipitation difference			Size during previous census		
Response	Estimate	SE	*P*-value	Estimate	SE	*P*-value	Estimate	SE	*P*-value	Estimate	SE	*P*-value
**A. Lifetime fitness**												
Lifetime fitness - zero inflation	0.025	0.124	0.840	**-0.342**	**0.033**	**< 0.001**	-	-	-	-	-	-
Lifetime fitness - conditional	-0.137	0.135	0.312	**-0.122**	**0.053**	**0.021**	-0.085	0.054	0.112	-	-	-
**B. Component lifestages**												
Germination	-0.024	0.085	0.776	**-0.100**	**0.034**	**0.003**	-	-	-	-	-	-
Size after germination	0.026	0.073	0.721	-0.027	0.026	0.302	-	-	-	-	-	-
Overwinter survival	0.229	0.127	0.072	**-0.087**	**0.032**	**0.007**	-	-	-	**0.367**	**0.033**	**< 0.001**
Size after winter	-0.061	0.176	0.728	**-0.515**	**0.095**	**< 0.001**	-	-	-	**0.748**	**0.042**	**< 0.001**
Fruit count	-0.009	0.155	0.954	-0.112	0.070	0.110	-0.099	0.063	0.115	**0.539**	**0.041**	**< 0.001**
Seed production	-0.101	0.134	0.448	-0.064	0.051	0.216	**-0.123**	**0.048**	**0.010**	**0.388**	**0.033**	**< 0.001**

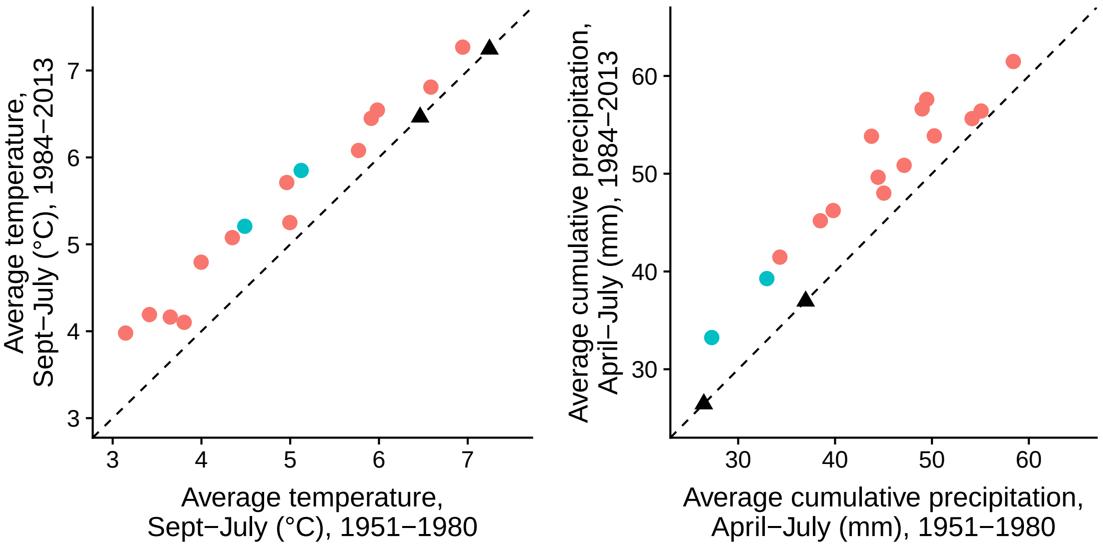

Figure S1 Correlation of temperature and precipitation normals calculated over the years 1951-1980 and 1984-2013. Each colored dot represents one source population, black dashed lines represent a 1:1 relationship, and black triangles represent conditions in the gardens during the experiment. Blue dots are focal populations, red dots are other populations. Climate data is from PRISM (PRISM Climate Group, 2017).

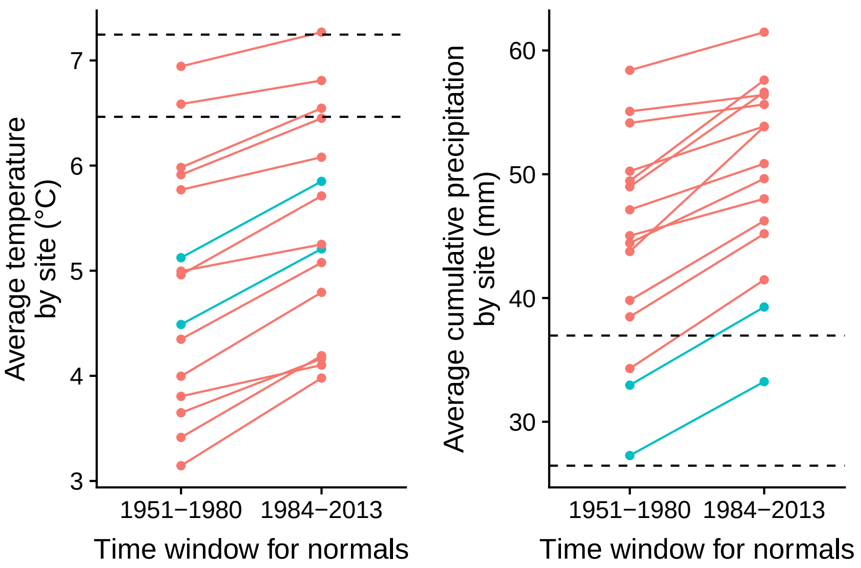

Figure S2 Temperature and precipitation normals calculated over the years 1951-1980 and 1984-2013, shown paired by site. Dashed black lines are the garden conditions. Blue dots are focal populations, red dots are other populations. Climate data is from PRISM (PRISM Climate Group, 2017).

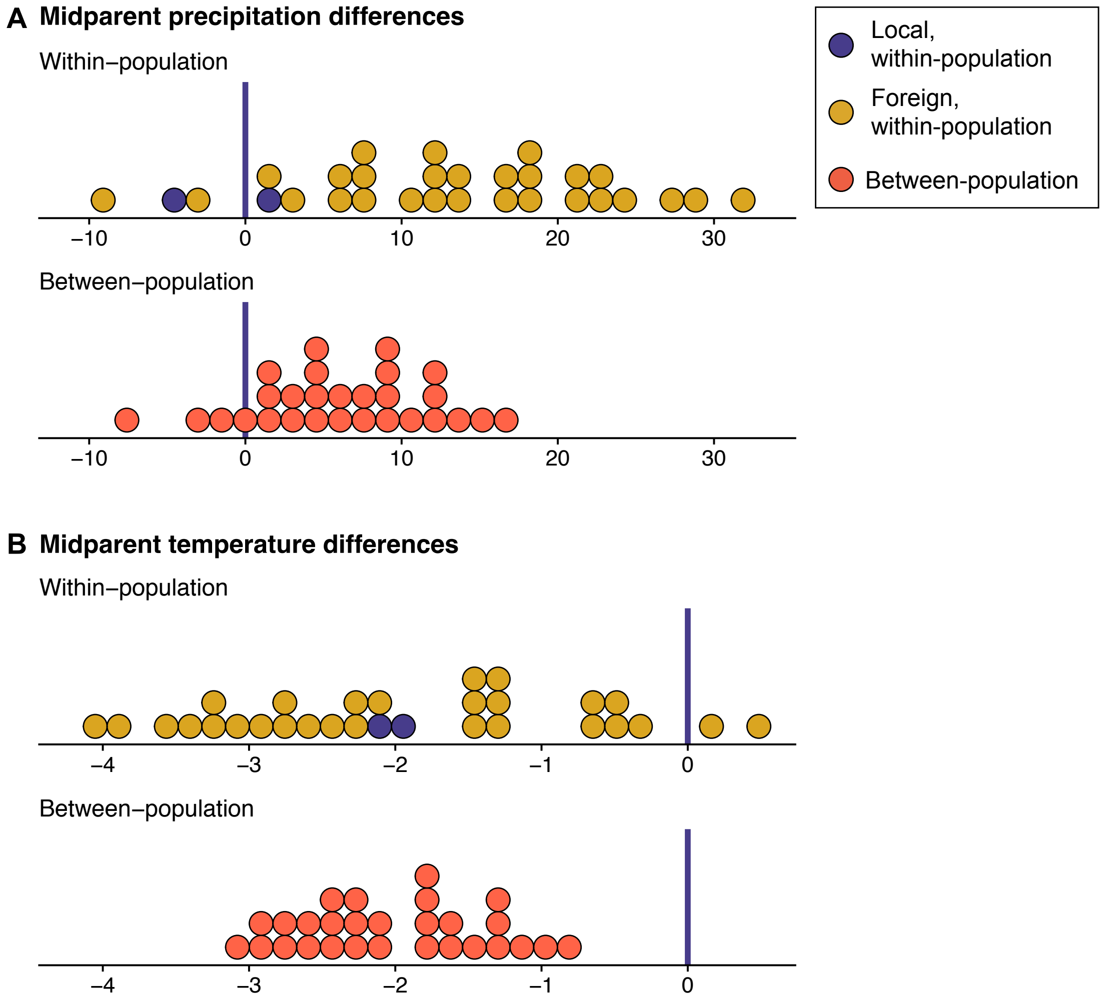

Figure S3 Distribution of climate differences of within- vs. between-population crosses of *Clarkia pulchella* relative to conditions during the experiment. Each dot represents a combination of maternal population, paternal population, and transplant site. Dark blue dots are within-population crosses of focal populations transplanted into their home sites. Gold dots are within-population crosses from donor populations planted into each of the two gardens, as well as the focal populations planted into each other's sites. Red dots are between-population crosses. Vertical blue bars are placed at zero, indicating where populations would be perfectly matched to the temperature or precipitation conditions during the experiment. **(A)** Distribution of the differences between the average temperature in the home sites of parental populations and conditions during the experiment. Focal populations are intermediate in temperature relative to other populations in the experiment; this results in similar average differences in temperature in between-population crosses and within-population crosses. **(B)** Distribution of differences between the average precipitation in the home sites of parental populations and conditions during the experiment. Focal populations are among the driest in the experiment; this results in smaller average differences in precipitation in between-population crosses compared to within-population crosses. Note that figures in the main text use absolute temperature differences: the absolute value of the midparent differences as they are plotted in this figure.

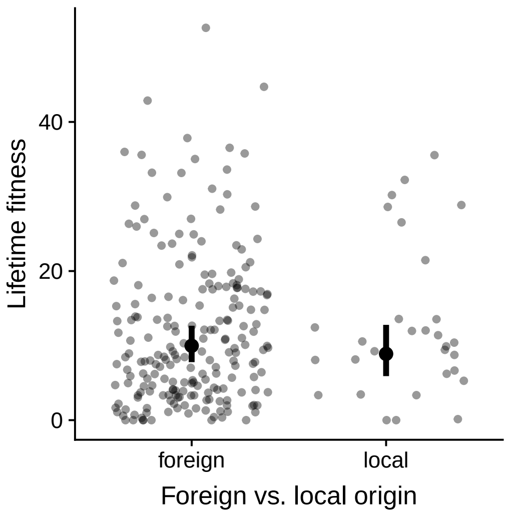

Figure S4 Lifetime fitness (seeds produced per seed planted) from populations of *Clarkia pulchella* with foreign vs. local parents. This analysis includes within-population plants only (no gene flow). Each point represents the average of a single family. Error bars are 95% confidence intervals of model estimated means, omitting variation from random effects.

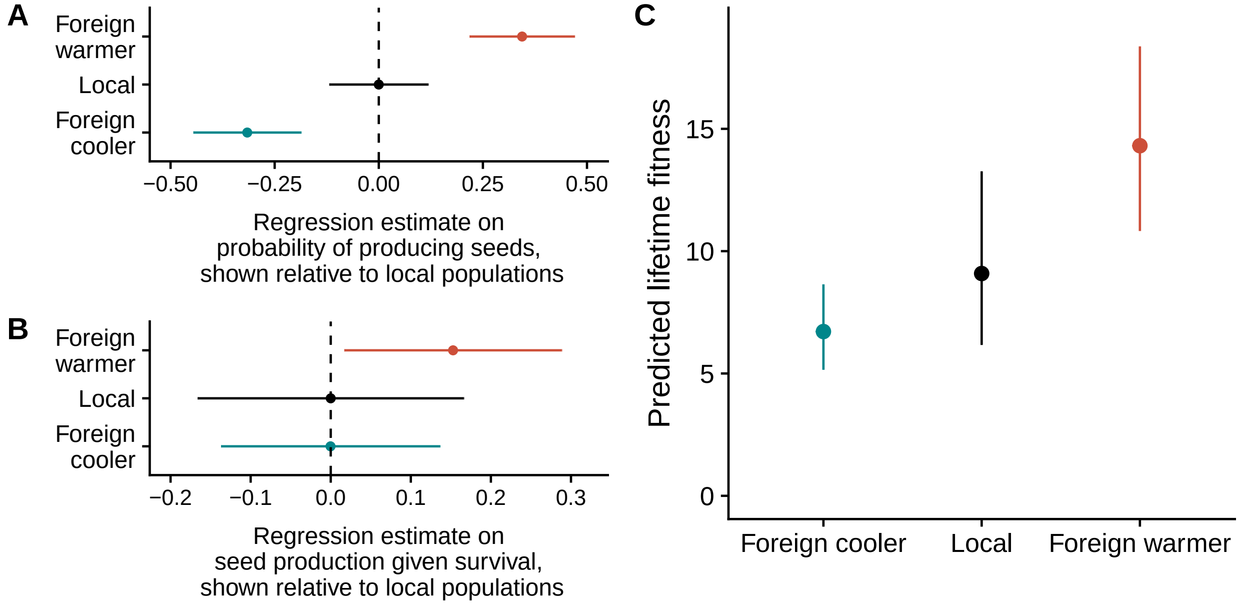

Figure S5 Regression estimates and predicted lifetime fitness of local populations (black) compared to foreign populations from warmer (red) and cooler (blue) provenances (Supplementary Analysis 1). **A** Estimated effects and standard errors of being from a warmer or cooler provenance on the probability of producing any seeds (the zero-inflation component of the model). **B** Estimated effects and standard errors of being from a warmer or cooler provenance on seed production, given survival (the conditional component of the model). Estimates in **A** and **B** are shown relative to local populations, which are set at 0. **C** Predicted lifetime fitness (seed produced per seed planted) of local and foreign populations from warmer or cooler provenances. These predictions integrate zero-inflation and conditional model components to give overall lifetime fitness projections (i.e. these predictions include seeds that did not survive to reproduce).

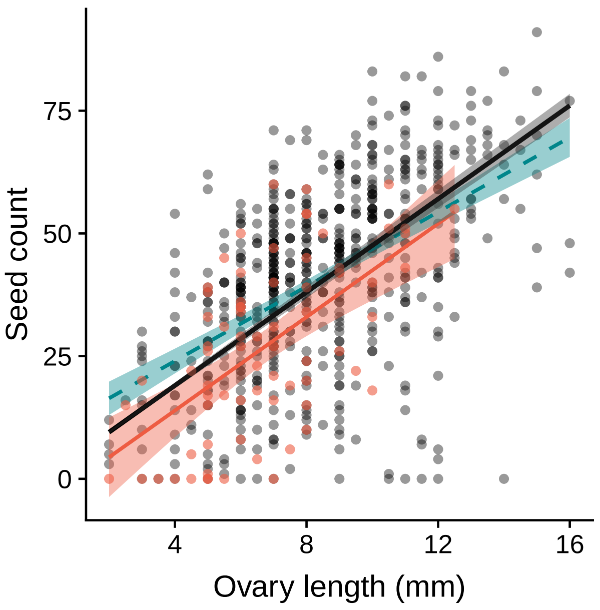

Figure S6 Immature ovary length (measured in the field) correlates with seed set after hand pollination (n = 596 fruits, grey points). This data was used to calibrate a conversion between ovary length in the field and potential seed production. The black regression line shows the relationship when the y-intercept is held at 0 (this line was used for our conversion); the dashed blue line shows the relationship when the intercept is not restricted. The red line and points show the relationship between ovary length and seed production in naturally occurring and naturally pollinated *Clarkia pulchella* in our common garden sites (n = 73 fruits).
